# Hints about Breakfast

**Published:** 1888-02-11

**Authors:** 


### HINTS ABOUT BREAKFAST.

FEW circumstances better illustrate and prove the essential conservatism of the English middle classes than their uniform and universal custom of eating bacon for breakfast. The pig, though a very unclean animal in a state of nature, has been vastly improved by civilisation—more perhaps than some of his masters—and is now as much a part of the culture and taste of the age as Liberty's silks or Mr. Grant Allen's agnosticism. Nevertheless, it is as possible to have too much pig as it is to have too much of Mr. Grant Allen's superiority and inconsequence. An occasional change for breakfast, extending even beyond the range of fish and kidneys, would be highly conducive to health, and serviceable as a means of retaining the palate in a high state of critical efficiency. The Americans have something to teach us in this respect. They do not think *bacon* the cardinal and fundamental article of breakfast-table faith. They even go outside the region of the animal world altogether, and substitute the produce of the cornfield in a variety of forms. The A B C breakfast cereals, of which samples have been sent to us by Mr. B. Lampe, of 44, Great Tower-street, are highly suitable for breakfast dishes for "all sorts and conditions of men"—including women. They consist of different forms of prepared white wheat, white oats, barley, and yellow maize. There is no doubt that all these cereals differ considerably from each other in ultimate composition, and that the occasional substitution of one for another in succession is of advantage to the digestion and general nutrition of the animal organism. We have personally tried them all, and can speak with confidence of their thorough palatability and wholesomeness. For children they are to be emphatically recommended; and as there is some want of variety in food for the senior nursery, mothers will do well to purchase samples, and prove for themselves how many different pleasant compounds can be made out of them. Invalids, who are always pleased with a new food, may try the A B C cereals without fear of dyspepsia or other unpleasant result. But whilst these foods are eminently suited to invalids and children, they are, as we have said, equally adapted to general use for adults of all ages and circumstances. If housewives could be persuaded to introduce them at the breakfast-table once or twice a-week, instead of the inevitable *Wiltshire* or *Yorkshire*, the pig might enjoy three or four months of added pleasure in the sty, and everybody's digestion and temper be correspondingly improved. It is proper to add that these cereals are decidedly cheap.

